# Children and providers’ perspectives on once-weekly rifapentine and isoniazid TB preventive therapy

**DOI:** 10.5588/ijtldopen.24.0250

**Published:** 2025-01-01

**Authors:** A.J. Marthinus, D.T. Wademan, Z. Saule, Y. Hirsch-Moverman, L. Viljoen, J. Winckler, L. van der Laan, M. Palmer, S.L. Barnabas, R. Boyd, A.C. Hesseling, G. Hoddinott

**Affiliations:** ^1^ Desmond Tutu TB Centre, Department of Paediatrics and Child Health, Faculty of Medicine and Health Sciences, Stellenbosch University, Cape Town, South Africa;; ^2^ ICAP at Columbia University, Mailman School of Public Health, New York, NY, USA;; ^3^ FAMCRU, Department of Paediatrics and Child Health, Faculty of Medicine and Health Sciences, Stellenbosch University, Cape Town, South Africa;; ^4^ Division of Tuberculosis Elimination, Clinical Research Branch, Centers for Disease Control and Prevention, USA;; ^5^ School of Public Health, Faculty of Medicine and Health, The University of Sydney, Sydney, NSW, Australia.

**Keywords:** paediatric TB, tuberculosis, LTBI, prevention, control programme, HIV

## Abstract

**BACKGROUND:**

TB preventive treatment (TPT) prevents the development of TB disease in individuals at risk of progression from infection to disease. However, implementation of TPT for children is poor in most high-burden settings. The long duration and pill burden of the 6-month once-daily isoniazid regimen (6H) pose significant barriers to completion. We aimed to understand children’s, caregivers’, and healthcare providers’ experiences of the 12-week once-weekly rifapentine and isoniazid (3HP) regimen using a dispersible tablet formulation in South Africa.

**METHODS:**

Serial, in-depth qualitative interviews with 20 child-caregiver dyads, including 5 children living with HIV (CLWH) and 9 healthcare providers across two study sites implementing a pharmacokinetic and safety trial of 3HP, were analysed deductively.

**RESULTS:**

Of those with experience using both 3HP and 6H, caregivers and healthcare providers preferred 3HP, and study participants reported that the 3HP formulation was more palatable and easier to prepare and administer. Caregivers and healthcare providers were concerned about optimally integrating 3HP into routine care, primarily due to its once-weekly administration. Children with HIV preferred the once-daily 6H regimen for its ease of use with their daily antiretroviral therapy.

**CONCLUSIONS:**

3HP reduced the administration burden for children and their caregivers. Once weekly, 3HP dosing will require education and adherence support to ensure completion.

## RÉSUMÉ

**CONTEXTE:**

Le traitement préventif de la TB (TPT) est essentiel pour empêcher l'évolution de l'infection tuberculeuse en maladie chez les individus à risque. Néanmoins, l'application du TPT chez les enfants est souvent insuffisante dans de nombreuses régions où la morbidité est élevée. La durée prolongée du traitement à l'isoniazide, qui nécessite une prise quotidienne pendant six mois (6H), ainsi que le nombre de comprimés requis, représentent des obstacles significatifs à son achèvement. Nous avons cherché à explorer l'expérience des enfants, des soignants et des professionnels de santé concernant le schéma de traitement combiné de rifapentine et d'isoniazide (3HP) sur une période de 12 semaines, en utilisant une formulation de comprimés dispersibles en Afrique du Sud.

**MÉTHODES:**

Des entretiens qualitatifs approfondis ont été réalisés avec 20 dyades composées d'enfants et de soignants, incluant 5 enfants vivant avec le VIH (CLWH) et 9 professionnels de la santé, dans le cadre de deux sites d'étude où un essai pharmacocinétique et d'innocuité de la 3HP était en cours. Ces entretiens ont été analysés selon une approche déductive.

**RÉSULTATS:**

Parmi les individus ayant une expérience avec la 3HP et la 6H, les professionnels de santé et les soignants ont manifesté une préférence pour la 3HP. Les participants à l'étude ont noté que la formulation de la 3HP était plus agréable au goût et plus simple à préparer et à administrer. Les soignants et les prestataires de soins de santé ont exprimé des préoccupations concernant l'intégration optimale de la 3HP dans les soins de routine, principalement en raison de son administration hebdomadaire. De leur côté, les enfants séropositifs ont montré une préférence pour le schéma 6H, qui implique une prise quotidienne unique, en raison de sa facilité d'utilisation en complément de leur traitement antirétroviral quotidien.

**CONCLUSIONS:**

La 3HP a réduit la charge administrative pour les enfants et leurs soignants. Une fois par semaine, l'administration de 3HP nécessitera une éducation et un soutien à l'observance afin d'assurer l'achèvement du traitement.

Children accounted for approximately 12% (approximately 1.27 million) of the estimated global TB cases in 2021.^1^ Children have a high risk of disease progression and are at risk of developing severe forms of TB following infection with *Mycobacterium tuberculosi*s (M.tb).^2^ TB preventive treatment (TPT) is an important strategy to reduce the burden of TB amongst children.^3^ However, only 55% of the 5-year target of 4 million children under 5 years old received TPT in 2018–2022.^1^

The completion of long TPT treatment courses (e.g., 6 months of daily isoniazid [6H], historically the TPT regimen recommended and widely used in high-burden settings) is low, especially among children living in high-burden settings.^4,5^ Implementation of TPT in high-burden settings such as South Africa remains sub-optimal, with only about 65% of eligible children initiating therapy and with completion rates as low as 50%.^6^ Additionally, the COVID-19 pandemic reversed progress in the South African TB programme, resulting in an estimated 1.4 million fewer people receiving TB care in 2022.^8^ In a review of TPT implementation in children in South Africa, we found multiple barriers at health systems and patient levels.^7^ Reasons for suboptimal implementation and uptake of TPT include health systems deficiencies, long duration of the regimens, misconceptions/perceptions of TB disease and preventive therapy and socioeconomic challenges, including stigma and health services costs.^9–11^

As of 2020, the WHO recommends shorter TPT regimens in children: 3 months of isoniazid (INH) and rifapentine (RPT) weekly (3HP) among children ≥2 years old or 3 months of daily rifampicin and INH (3RH).^3^ The 3HP regimen has proven efficacy, with fewer adverse effects and higher treatment completion rates than the 6H and 9H regimens.^12,13,14^ Children <2 years old are currently excluded from the 3HP recommendation as no pharmacokinetic and safety data is available in this group. Tuberculosis Clinical Trials Consortium (TBTC) Study 35 is designed to determine the pharmacokinetics and safety of 3HP in children aged 0–2 years and older with and without HIV (NCT03730181). The trial used non-commercial paediatric dispersible RPT formulations, including a 150 mg RPT and 150 mg INH (1:1) fixed-dose combination formulation and a 100 mg RPT standalone formulation (both Sanofi-Aventis, Bridgewater, NJ, USA).

In addition to the need for dosing and safety data in young children, there are gaps in our understanding of children’s and caregivers’ experiences and acceptability of the 3HP regimen and dispersible formulations. Predominant definitions used for the acceptability of treatment in children focus on the overall ability of the ‘user’ to use the therapy as intended.^15^ Underlying this definition is the recognition that poor acceptability will impact adherence and, therefore, the efficacy of TPT. Psychosocial factors, including stigma, loss of agency and fear, can also negatively impact the acceptability of treatment.

We conducted a qualitative sub-study nested in TBTC Study 35 to understand children’s, caregivers’ and healthcare providers’ perceptions and experiences of the 3HP regimen. Specifically, we explored their experiences preparing, ingesting, or administering the 3HP regimen using the novel dispersible formulation.^16^

## METHODS

### Setting

South Africa had an estimated TB incidence of 468 notified cases per 100,000 population in 2022.^17^ The study was implemented at two sites in Cape Town, South Africa.

### Study design

We completed a case descriptive study with longitudinal qualitative data. Participants in TBTC Study 35 were children aged 0–12 years, including children living with HIV (CLWH) and without, who had been exposed to an adult household contact with infectious drug-susceptible TB in the past 6 months (sputum culture-confirmed or Xpert^®^ MTB/Rif Ultra (Cepheid, Sunnyvale, CA, USA) positive TB without evidence of drug resistance) or who had a positive test (positive TB skin test ≥10 mm in HIV-negative and tuberculin skin test ≥5 mm in HIV-positive participants or a positive commercial interferon-gamma release assay) of TB infection. All children received 12 once-weekly doses of water-dispersible fixed-dose combination formulations of RPT and INH.^18^ The dispersible formulation was prepared and administered by the study team at the research site during scheduled study visits. Additional RPT was given to children in accordance with WHO weight bands. Caregivers, children, and healthcare providers were interviewed to understand their perspectives on the 3HP regimen.

### Sampling

Purposive, maximal variation sampling ensured the recruitment of a heterogeneous sample of child-caregiver dyads to achieve diversity in children’s age, sex, and HIV status. We also recruited healthcare providers at respective trial sites to understand their experiences with trial participants on the novel 3HP formulation.

### Data collection

Data collection took place between February 2021 and August 2022. We conducted a series of audio-recorded in-depth interviews (approximately 3 per child-caregiver dyad) over the course of the child’s 3HP regimen. We also interviewed healthcare providers at the trial start and approximately 18 months later. Data were collected by three socio-behavioural scientists (AM, ZS and DW) under the supervision of GH and LV.

In response to the Covid-19 pandemic, South Africa imposed a hard lockdown in March 2020, with most clinical research activities suspended until September 2020. As a result, nine child-caregiver dyads were recruited after completing the trial. For the remaining participants, the first two interviews were conducted at Weeks 2 and 6 of the study, to ensure optimal recall of 3HP preparation and administration. The final interviews were conducted after treatment completion in Week 12 at the participant’s household to contextualise the interpretation of their socioeconomic context.

All interviews with child-caregiver dyads followed three thematic areas: 1) caregivers’ and children’s knowledge, perceptions, and understanding of TB disease and treatment, including TPT, 2) participants’ experiences of 3HP in the context of the trial, and 3) understanding participants’ socio-economic context. We used participatory, child-centred research activities during the interviews with the child-caregiver dyads to stimulate conversations about each thematic area.^19–21^ Interviews with healthcare providers provided insights and perceptions of potential barriers and facilitators to implementing TPT at their facility. Interviews ranged from 30 min to 2 h and were conducted in participants’ preferred language (English, Afrikaans or isiXhosa).

### Data analysis

After each interaction with child-caregiver dyads and healthcare providers, the interviewing researcher completed a case description detailing participants’ experiences as reported in the interview, noting key verbatim quotes and reflecting on the researchers’ observations. The larger research team reviewed each case description, and additional supplementary details were added in response to this iterative analytic review. We used the domains of usability and receptivity from Wademan et al.’s conceptual model of the acceptability of TB care for children as an analytic frame for the deductive analysis.^16^

### Ethics statement and informed consent

The study was approved by the Health Research Ethics Committee at Stellenbosch University (N20/09/093) and the US Centers for Disease Control and Prevention (CDC) Institutional Review Board. Written informed consent was obtained from all caregivers, including themselves, their children, and participating healthcare providers. Written informed consent was obtained from children 7–12 years of age.

## RESULTS

We present findings from 56 interviews with 20 caregivers of 23 children. The median age of the children was 5 years, with an interquartile range of 1.1 to 10 years. The ages of the child participants ranged from 0.25 to 12 years. Nine children had initiated 6H before being referred to and enrolled in the trial and, therefore, had direct experiences to compare between regimens. Five CLWHs were included. Interviews (*n* = 14) were conducted with nine healthcare providers involved in the trial to include their perceptions of the 3HP regimen.

### Children, caregivers’, and healthcare providers’ experiences of drug administration and adherence support practices

Caregivers whose children were being treated with the once-daily isoniazid on the 6H regimen and healthcare providers perceived the 3HP regimen to be easier to prepare and administer. Children were not averse to the taste of the 3HP formulations, and caregivers found that it was easy to administer, with only minor challenges reported, such as taste and palatability.

### Palatability

A common perception among caregivers was that their children liked the taste of the 3HP formulations; they tasted sweet and like fruit, predominantly mango flavour. One healthcare provider said, ‘You can smell the mango as soon as you put [the pills] in the water.’ The caregiver of a 9-month-old boy described her son’s reaction when taking the treatment for the first time, ‘he drank this one. He was not fussy, he drank it’. Conversely, she reflected on her previous experience of administering 6H as ‘it has a bitter taste [and] he did not want it. I would force him every day’.

However, there were a few conflicting experiences with 3HP. A 12-year-old boy said: ‘Gosh! It tastes so bad!’. Later, he recalled vomiting a few hours after taking the treatment on his way home. This only happened at treatment initiation and may have been influenced by other external factors.

A caregiver of a 3-year-old boy described how her child would refuse the treatment every time they came to the clinic, saying:

…he still doesn’t want to drink it. He spits it out […]. Then he keeps his mouth closed. Then he’ll try some then spits it out […] I don’t think anything would make the process easier.

Similarly, healthcare providers reported that some children would pull a face when taking treatment, suggesting that it was due to the poor palatability of the formulation.

### Practical administration

Caregivers who collected the 6H regimen from their local clinic mentioned difficulties with administering the tablets when asked about challenges with TPT. In the trial, 3HP is prepared as dispersible tablets dissolved in water and administered by the study team. Healthcare providers described the 3HP preparation as quick and easy, taking less than 15 min from preparation start to administration. One healthcare provider explained,

It’s not difficult. You just measure the amount of water, put the tablet in the cup until it dissolves. Wait for a few minutes, just swirl it.

Although the caregivers were not directly involved in preparing and administering the 3HP regimen, they observed this process and reported that it was easier than their experiences preparing and administering 6H at home. Children and caregivers described healthcare providers’ treatment preparation process as simple and easy to follow. For example, a 12-year-old boy recalls: ‘They first crush it, then pour in water and tell me to drink quickly.’ This participant likely assumed the treatment was crushed first due to how quickly it dissolved. A mother of a 1-year-old girl compared her administration experience of the 6H regimen to that of the 3HP regimen, saying,

…that everyday pill [6H] is difficult to give because I had to buy yoghurt to improve its taste and to make it easier to administer, and then [the 3HP] became once a week.

The same caregiver described that her niece was on the 6H regimen while her daughter was on the 3HP regimen. She continued:

It’s very difficult like my sister’s child [aged 4] still throws up the tablets [6H] even in the yogurt and spits it out. It makes her nauseous, but with this one [3HP regimen], it’s easier.

Caregivers, especially those of young children, said their children preferred to drink the treatment in a liquid form rather than tablets, which sometimes needed to be crushed and sprinkled in yoghurt or other food. For example, a caregiver of a 4-year-old boy said:

No, they did not administer it in a bad way because he would at least drink it when they prepare it. So, I think at least there [at the clinic] he knew he drinks the medicine on his own. [...] No, it is all right as it is, because at least it tastes good.

Although some participants preferred the 3HP regimen mixed with water, other caregivers and children (especially older children) said they would have preferred to swallow the tablets whole.

As a caregiver of an 11-year-old boy who was also on antiretroviral therapy (ART) said: ‘I would give him the pills because he prefers them’. Conversely, a caregiver of an 8-month-old girl said that she would prefer the treatment to be a pre-prepared syrup/suspension: ‘because I would not be required to mix anything together.’

### Adherence support practices

A major contributing factor to caregivers’ and children’s preference for the 3HP over the 6H regimen was the shorter regimen duration. However, participants’ opinions about being able to remember once-weekly dosing for 3HP were mixed.

Some caregivers acknowledged they had missed dosing their children while they were on 6H. When asked about their capacity to administer once-weekly doses of 3HP, caregivers noted that they would easily remember and be able to integrate administration into their routine. One caregiver of three children (a 12-year-old boy and 6-year-old twins) said that she would have preferred to administer the 3HP regimen at home rather than attending the clinic every week: ‘There would be no challenges, I would administer it how they do it [at the clinic]’.

While caregivers said the once-weekly dosing of 3HP would not be difficult, caregivers also said that they would prefer to go to the clinic where a nurse could help administer the treatment to their child to ensure their children were dosed correctly and not have to worry about forgetting to administer any doses. CLWH and their caregivers found daily treatment adherence easy to integrate into their existing care practices. To manage the weekly dosing of 3HP, the caregiver of a 12-year-old boy on ART said she would store the 3HP next to her child’s other treatment. The mother explained,

...all you have to do is you just put that pill [3HP] by those pills [ARVs] which he drinks every day. So, as you pick up those tablets, then you’re going to pick up this bottle as well […] I would just set a reminder to say Tuesdays, he must drink that tablet.

Caregivers and healthcare providers raised concerns regarding the possibility that caregivers might forget to administer a once-weekly dose. Some healthcare providers expressed this concern more frequently. However, healthcare providers with this concern also said that caregivers would manage as long as they were provided with sufficient information on treatment preparation and support. A healthcare provider explained: ‘It’s just the principle of, you know, engaging with people and educating them about how medication is supposed to be taken’. Healthcare providers’ concerns around 3HP adherence had more to do with how it would be rolled out in the public health system. One healthcare provider shared their concerns about non-adherence, suggesting that:

If the [caregiver] is reliable, maybe they can give the first doses at the clinic […] the other doses the [caregiver] gives at home, so they see her [at the clinic] once every three weeks. But if it’s a [caregiver] they’re unsure about, then she should rather come weekly […]. But there’s also the option of DOTS [*sic. It is likely the participant meant direct observation therapy*] workers going to dose. [..] It’s important that there should be close follow-up, and even a reminding call to remind the [caregiver] that ‘today your child needs to take their once weekly 3HP dose’, and then a support line.

Healthcare providers were, therefore, more concerned about caregivers’ ability to routinely access 3HP, differentiate its dosing from other drugs, and receive support throughout the treatment period to reduce the burden of care added by 3HP.

### Changes in experiences over time

Caregivers and children who reported that they did not initially like to take/administer the 3HP nor enjoyed the taste became familiar with the treatment administration process over time. For example, a 12-year-old girl noted that she became used to the taste of treatment as time passed, saying, ‘It was not nice at the beginning, but it is better now’. Healthcare providers also reported that some children became accustomed to the taste over time. For example, a healthcare provider said:

It’s not like the kids are spitting it out […] it’s not that horrible […] they get used to it […] by the third or fourth visit to the clinic.

While it was not common, some children continued having unpleasant experiences. For example, the 12-year-old boy who initially said 3HP tasted bad later said: ‘It makes me nauseous sometimes. Not all the time, but I drink it. It’s not nice. […] it’s normal to take the medication’.

The caregiver of a 3-year-old boy whose treatment was administered through a nasogastric tube after multiple failed attempts at administration explained that her child rejected all treatments and not the 3HP regimen specifically. In addition, the caregiver admitted that if she had to administer the medication herself at home, it would be problematic, saying: ‘No, if my child doesn’t want that [treatment], then I won’t give it’. This child and caregiver’s experience suggests that distal factors not related to the treatment formulation may influence overall acceptability.

## DISCUSSION

Children and caregivers with experience of both 6H and 3HP preferred the taste of the 3HP regimen over the 6H monotherapy regimen. Healthcare providers and caregivers with experience with both 6H and 3HP regarded the 3HP regimen as easier to prepare and administer. Although most children had little to no difficulty ingesting the treatment, some may experience difficulty not related to the medication itself but to the behavioural process (e.g., fighting; hiding; running away; spitting the medication out) involved when taking treatment. This could indicate that the taste of treatment could still be improved as children may be less resistant to taking the medication if the flavour was improved. There were some concerns by healthcare providers about integrating 3HP into routine care, primarily due to its once-weekly administration schedule, which could lead to forgetting doses. While there were caregivers who felt that administering the medication at home would be easy, other caregivers suggested going to their local clinic to receive the once-weekly 3HP regimen to prevent non-adherence. Healthcare providers suggested that 3HP be rolled out with intensive education and support structures to assist caregivers and reduce the possibility of non-adherence.

Strengths of our study include in-depth data collected longitudinally over the course of the 3HP treatment period, including children themselves as well as their caregivers, and including a diverse sample with regard to age, sex, HIV status and language. Since some participants had prior experience with 6H administration, they could also qualitatively compare 3HP to 6H. Limitations include the fact that sub-group analyses were not possible due to modest sample sizes. Further, caregivers only observed 3HP dose preparation by study staff, which was simple and easy to do since they did not prepare doses themselves. Although healthcare providers’ experience of TB (and other disease prevention) was broad, their close involvement in the trial could have made them biased towards the treatment (or biased in favour of the 3HP regimen). Further data on using this regimen in routine care for children would be valuable.

Shorter regimens, such as 3HP and 3HR, could significantly increase the uptake and completion of TPT among children.^22,23^ Studies reporting on shorter TPT regimens have focused on efficacy, tolerability, safety and adherence, with little reported on its acceptability amongst participants, including children and caregivers.^13,24,25^ Our study addresses this key gap. Further, dose preparation and administration processes play an integral role in the overall acceptability of TPT in children.^26,27^ TPT preference studies found that shorter TPT regimens, dosing frequency and child-friendly regimens were important preference characteristics in Peru,^28^ Lesotho^22^ and South Africa.^29^ Ingestion of medication may pose a barrier to younger children, especially if caregivers struggle to administer medicines to their children and indicate that caregivers play a crucial role in the uptake of treatment.^22,30^ In our study, caregivers of younger children experienced fewer (compared to 6H) medication administration challenges. Participants preferred 3HP because of the shorter treatment duration, ease of dose preparation, and palatability of the formulation.

Similar to findings from a TPT study in Peru,^28^ caregivers and healthcare providers in our study also preferred shorter regimens and child-friendly formulations even though healthcare workers felt that the taste of 3HP could still be improved. Some caregivers in our study were worried they might forget doses on a once-weekly schedule. Some caregivers mitigated that challenge by suggesting receiving treatment at the clinic if it was administered once weekly. However, it is also important to note a difference from the study in Peru: participants in our study were not concerned about the adverse effects of a once-weekly regimen. Children’s acceptability of TPT regimens may differ by HIV status and other factors such as age and prior treatment experience, among others, indicating the value of giving caregivers an option on which regimen to use for TPT.

Our findings add to growing evidence on the acceptability and local social value of shorter TPT regimens, specifically 3HP, in children. Policy guidelines and routine practice should consider this evidence alongside data on dosing, safety, and efficacy data to accelerate children’s access to the 3HP regimen. Future research should prioritise the evaluation of acceptability and adherence support in multiple settings as 3HP becomes available to children in routine care in future, including the use of commercially available formulations.
